# Viscoelastic, Optical, and Surgical Properties of Vitreous Body Replacement Hydrogels After Aging Compared to Porcine Vitreous Bodies And Silicone Oils

**DOI:** 10.1167/tvst.13.7.5

**Published:** 2024-07-05

**Authors:** Maximilian Hammer, Marcel Muuss, Lorenz Herbster, Jonathan Herth, Alexander Scheuerle, Ramin Khoramnia, Grzegorz Łabuz, Philipp Uhl, Gerd Uwe Auffarth

**Affiliations:** 1 David J Apple Laboratory for Vision Research, Heidelberg, Germany; 2 University Eye Clinic Heidelberg, Heidelberg, Germany; 3 Institute for Pharmacy and Molecular Biotechnology, Heidelberg, Germany

**Keywords:** vitreoretinal surgery, vitrectomy, vitreous humor, silicone oil

## Abstract

**Purpose:**

First- (monomers), second- (pre-gelated), and third- (in situ gelating after injection) generation hydrogels were previously introduced to replace the vitreous body after vitrectomy surgery. In this study, we evaluated the surgical, optical, and viscoelastic properties of vitreous body replacement hydrogels before and after an accelerated aging protocol previously applied to intraocular implants.

**Methods:**

Measurements of injection force, removal speed using a clinically established vitrectomy setup, as well as evaluation of forward light scattering and viscoelastic properties before and after an accelerated aging protocol were conducted. Results were compared to porcine and human vitreous bodies, as well as currently clinically applied lighter- and heavier-than-water silicone oils.

**Results:**

Removal speed of all tested hydrogels is substantially lower than the removal speed of porcine vitreous body (0.2 g/min vs. 2.7 g/min for the best performing hydrogel and porcine vitreous body, respectively). Forward light scattering in second-generation vitreous body replacement hydrogels was higher after the aging process than the straylight of the average 70-year-old vitreous body (9.4 vs. 5.5 deg^2^/sr, respectively). The viscoelastic properties of all hydrogels did not change in a clinically meaningful manner; however, trends toward greater stiffness and greater elasticity after aging were apparent.

**Conclusions:**

This study demonstrates surgical weaknesses of the hydrogels that need to be addressed before clinical use, especially low removal speed. Pre-linked hydrogels (second-generation) showed inferior performance regarding surgical properties compared to in situ gelating hydrogels (third-generation).

**Translational Relevance:**

This study highlights possible pitfalls regarding surgical and optical properties when applying vitreous replacement hydrogels clinically.

## Introduction

For all surgical interventions involving retinal tissue, the vitreous body must be removed from the vitreous cavity. At the end of especially retinal detachment surgery, an endotamponade is placed inside the eye to stabilize the retina. Currently, clinically used endotamponades are air^1^ or other gases and balanced salt solution, as well as silicone oils of varying density.^2^^,^^3^ The oil's properties regarding their surgical performance,^4^^,^^5^ physicochemical behavior,^6^ and influence on pharmacokinetics within the eye^7^ have been intensely studied, leading to major improvements of this tamponade in the past. The addition of high-molecular-weight polydimethylsiloxane improved the tendency of the oil to emulsify.^8^ However, many of the drawbacks of silicone oils, like their adhesion to intraocular lenses or the tendency to emulsify in the vitreous cavity leading to secondary inflammation and intraocular pressure spikes, are based on its lipophilic character.^9^^–^^11^ Thus, during the last decades, hydrophilic vitreous body replacements were developed as substitutes for the vitreous body and directly compared to silicone oils in animal studies, often outperforming the silicone oil.^12^

These hydrophilic vitreous body replacement strategies can be categorized into four categories ranging from uncross-linked polymers^13^ through preformed chemically crosslinked hydrogels^14^^–^^16^ to in situ chemically crosslinked^17^ and physically gelating hydrogels. The first generation of hydrogels are uncross-linked monomers.^18^^,^^19^ Because of their short residence time, high swelling pressure, and bad tamponading ability,^20^^–^^23^ this approach has been left for vitreous body substitution purposes and is only applied during anterior segment surgery.^24^^,^^25^

The second generation of hydrogels refers to preformed chemically cross-linked hydrogels. However, the preformation is problematic because during the injection procedure forward light scattering is increased in these hydrogels because of the gel's fragmentation.^26^ Additionally, biocompatibility might be an issue due to remnants of the cross-linking solutions. However, a wide variety of cross-linking reaction and monomers has been proposed in the past, including alginate, hyaluronic acid,^14^^–^^16^^,^^27^^,^^28^ polyethylene glycol,^29^ and tetra-polyethylene glycol.^17^

The third generation of vitreous body replacement hydrogels prevents gel fragmentation with compromised optical clarity by gelating in situ. This allows the combination of many positive effects; however, it is essential that the monomers are tested for toxicity in detail because the reaction occurs within the eye.^17^ Multiple reaction, including click chemistry,^17^ aldehyde condensation,^30^ and Schiff base^31^ reactions were suggested.

Next to viscoelastic properties, as previously mentioned, ophthalmological implants are required to have favorable optical properties. One important factor is forward light scattering, the amount of light scattered at finely dispersed impurities in a forward direction and therefore being projected onto the retina. This parameter was previously mostly applied to study intraocular lens complications,^32^ such as the calcification of hydrophilic polymers^33^ or the occurrence of glistenings in hydrophobic polymers.^34^ Recently, our group also applied it to the vitreous body^35^ and vitreous body replacement hydrogels for the first time.^26^ However, many of these optical complications only occur after a longer duration within the eye. It is therefore common practice for ophthalmological implants, such as intraocular lenses (IOLs), to undergo stress testing by accelerating aging using high temperatures.

The optical and viscoelastic properties of proposed hydrogels to replace the vitreous body have never been investigated after stress testing. Thus we applied a commonly applied IOL-aging protocol^34^ to a variety of vitreous body replacements strategies and evaluated viscoelastic and optical parameters before and after aging in comparison to results of porcine vitreous bodies and commonly used silicone oils.

Additionally, although prior studies have tested hydrogels independent of each other in a small subset of in vivo experiments, none focused specifically on the surgical properties, such as force needed to inject the gels or the gel's ability to be extracted from the vitreous cavity in case of follow-up retinal surgery being necessary. Thus we evaluated the surgical properties, namely the needed injection pressure and the removal speed, of a variety of hydrogel suggested for vitreous body replacement.

## Material and Methods

### Materials

Siluron 5000 and Densiron 68, lighter- and heavier-than-water silicone oils (both Fluoron GmbH, Ulm, Germany) were examined in this study. As the first-generation vitreous body replacement hydrogel, the OVD Pe-Ha-Luron F 1.0% (Albomed GmbH, Schwarzenbruck, Germany) was used. Furthermore, alginate solution (0.5% and 0.65% Alginatec GmbH, Riedenheim, Germany) and 4ARM-SH-10K (M = 10 kg/mol) and 4ARM-MA-10K (M = 10 kg/mol) were used for this study (both by JenKem Technology, Tianjin, China).

### Second-Generation Tetra-PEG Gel Synthesis

A second-generation Tetra-PEG hydrogel was formed by cross-linking an oligo-Tetra-PEG solution with DL-Dithiothreitol (DTT) (Sigma Aldrich, Missouri, USA) as first presented in Hayashi et al.^17^. The oligo-Tetra-PEG solutions were prepared at c0 = 60 g l−1 and r = 0.13 in CPB (pH 5.8, salt concentration 5.0 mM) with NaCl (149 mM). DTT was dissolved in CPB with NaCl in a quantity matching the number of unreacted functional groups in the oligo-Tetra-PEG-solution and after 12 h mixed with the oligo-Tetra-PEG solution, resulting in a gel at c = 13.5 g l−1.

### Second-Generation Alginate Gel

Five milliliters of alginate solution (either 0.5% or 0.65%; Alginatec, Riedenheim, Germany) were injected into a tubular dialysis membrane (8 kDa, Ø 11.5 mm; Spectra/Por 7 Dialysis Membrane; Repligen, Boston, MA, USA), sealed and placed into an aqueous 11.6 mM calcium sulfate dihydrate solution for cross-linking at room temperature for at least four hours as previously described.^16^^,^^36^

### Third-Generation Tetra-PEG Gel Synthesis

As introduced by Hayashi et al.^17^, two pre-gels were prepared. Respectively, solutions of 12.6 g/L and 7.4 g/L of both, 4ARM-SH-10K and 4ARM-MA-10K, with citrate-phosphate buffer (pH 5.0, di-sodium hydrogen phosphate dihydrate and citric acid monohydrate) were created. Mixtures of equal volumes of each higher concentration solution and lower concentration solution were produced and left for at least 12 hours, aiming to reduce the in situ gelling time through the creation of oligo-Tetra-PEGs. After the creation of Oligo-Tetra-PEGs, equal amounts of high-SH solution and high-MA solution were mixed to produce the crosslinked gel in sample glasses or, for the forward light scattering measurements, in UV cuvettes.^2^ The cross-linking occurs without catalysts within a few minutes through the thiol-maleimide-click-reaction.^37^

### Accelerated In Vitro Aging Procedure

Based on the accelerated aging protocol for IOLs by Thomes et al.,^38^ already applied for IOLs by the David J Apple Laboratory,^39^ a similar approach was conducted in this study. All substances were sealed and incubated at 45°C for 24 hours using a water bath. In contrast to the previously applied protocol, which conducted all measurements at 37°C, samples were cooled down to 20°C for measurements, because this temperature is considered standard for viscoelastic measurements.

### Vitreous Body Preparation

Fresh porcine eyes of pigs aged eight to nine months were purchased from a local slaughterhouse (Schradi Frischfleisch, Mannheim, Germany) and transported to Heidelberg University (<1 hour). An incision was made at the equator to obtain the vitreous body, which was gently freed of any remaining pigment and retinal debris using forceps and scissors and rinsed with balanced salt solution (BSS). It was placed directly into the optical measurement cuvette or sample glass.

### Forward Light-Scattering Measurements

For the measurement of straylight a C-Quant device (Oculus, Wetzlar, Germany), which serves to evaluate ocular straylight clinically, was modified by our group and already applied to study the straylight of intraocular lenses or vitreous body replacement hydrogels.^26^^,^^32^^,^^40^^,^^41^ The C-Quant functions based on a psychophysical compensation comparison method. To enable a repeatable and objective evaluation of straylight a custom-made optical mount was added to the C-Quant. It is based on a field-stop, which enables the observer to judge the light scattered by the sample only. Each substance was measured three times in a row.

### Injection Pressure

The resistance force during the injection of 2 ml of each substance was measured and evaluated using an automated digital force gauge meter FV-10XY (Shimpo Instruments, Linbrook, NY, USA) with the Shimpo Toriemon Force Gauge Software. These measurements were only conducted before aging, and for each substance both a 23G and 25G cannula with, respectively, three measurements, was used. For the cross-linked hydrogels, the substrates were directly mixed within the 10 mL syringe and the measurements were performed immediately in a row. Changes in the resistance force during injection are shown as a curve in a graph, and the maximum peak of each curve in the graph was evaluated.

### Vitrectomy

For in vitro evaluation of removal speed during vitrectomy of the different substances, the microsurgical system Megatron S3 (Geuder AG, Heidelberg, Germany) was used with a 23 gauge/0.6 mm vitrector, 800 cuts/min and a vacuum of 600 mm Hg. Respectively, about 5 mL of the sample were placed in a petri dish, weighted, and three times in a row vitrectomized for one minute with subsequent weighing. The vitrector was gently moved through the substance during the vitrectomy process.

### Measurement of Viscosity and Viscoelastic Properties

About 0.6 mL of each substance was used for the measurements of viscosity and the viscoelastic properties with the rheometer (Anton Paar MCR, 302e; Anton Paar, Graz, Austria). The samples were placed in the middle of the lower rheometer plate and then the second plate was lowered to a distance of 1 mm. Frequency sweeps were performed from 1 to 10 rad/s. Measurements were conducted at 20°C. For the hydrogels and porcine vitreous bodies, roughened plates were used to prevent wall slip.^42^

### Statistical Analysis

Prism 10 (GraphPad, San Diego, CA, USA) was used for all statistical analyses. Normality was assessed and statistical tests chosen appropriately.

## Results

### Surgical Evaluation

#### Injection Pressure

**Figure 1.**
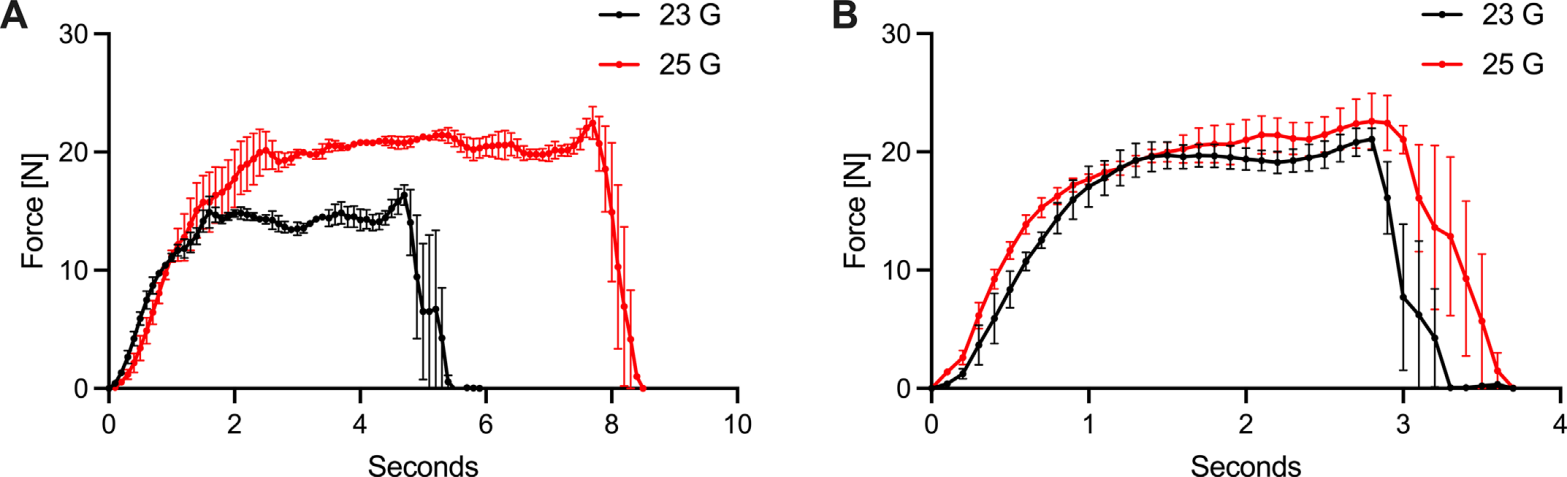
Exemplary pressure curves for a first-generation hydrogel (**A**) and a third-generation hydrogel (**B**) at 23 and 25 gauge. Mean ± SEM, all measurements were conducted in triplicates.

**Figure 2.**
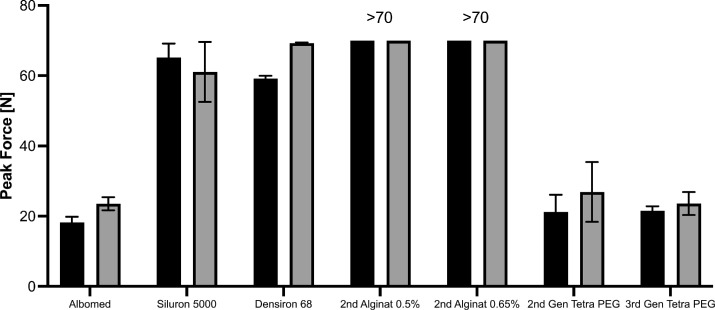
Quantitative analysis of peak force. The third-generation hydrogel (Tetra-PEG) showed superior injection properties as gelation occurs in situ (N = 3; >70 indicates that the measurement exceeded the measurement range of the force gauge meter).

Most hydrogels, including even the preformed second-generation Tetra-PEG hydrogel, outperformed the high viscosity lighter-than-water silicone oil regarding the needed injection pressure. third-generation hydrogels showed distinct advantages over second-generation hydrogels regarding the ease of injection (peak pressure, *P* < 0.0001, see Fig. 2). No significant difference between the peak force of first- and third-generation hydrogels was detected. First-generation gels are currently used in anterior segment surgery and are injected by hand indicating easy surgical handling. Figure 1 shows exemplary injection pressure graphs at 23G and 25G for the first- and third-generation hydrogels. Figure 2 depicts quantitative analysis of the peak pressure used during the injection process.

#### Removal Speed

**Figure 3.**
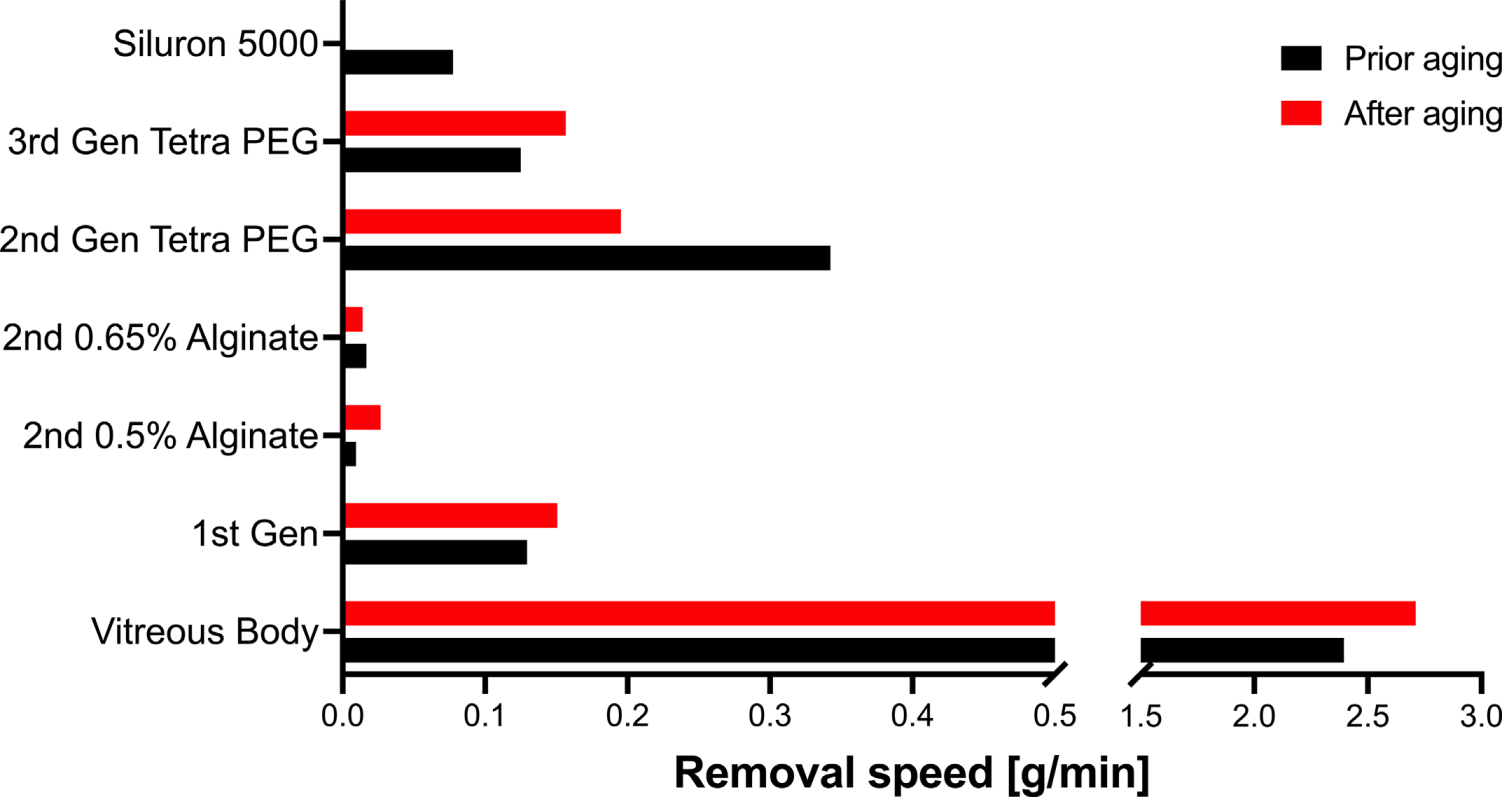
Removal speed for all tested hydrogels compared to silicone oil and the porcine vitreous body. The hydrogels showed distinct removal speeds. Hydrogels based on Tetra-PEG showed removal speeds superior to silicone oils. The Siluron 5000 result is based on data published previously by Hammer et al.^5^ using an optimized setup for silicone oil removal and not the vitreous cutter. The oil cannot be removed with the vitreous cutter. Compared to the vitreous body, removal speeds of the hydrogels were surprisingly low (N = 3).

All hydrogel replacement strategies showed a majorly reduced removal speed with a conventional 23G vitreoretinal cutter in comparison to the porcine vitreous body or balanced salt solution. Interestingly, one of the tested second-generation vitreous body replacement strategies could not be removed using the current surgical standard. No significant difference was found for any of the hydrogels when comparing the removal speed before and after the application of the aging protocol. Figure 3 depicts removal speeds (Removed weight in grams/min) before and after the aging protocol.

### Aging Evaluation

#### Forward Light Scattering

**Figure 4.**
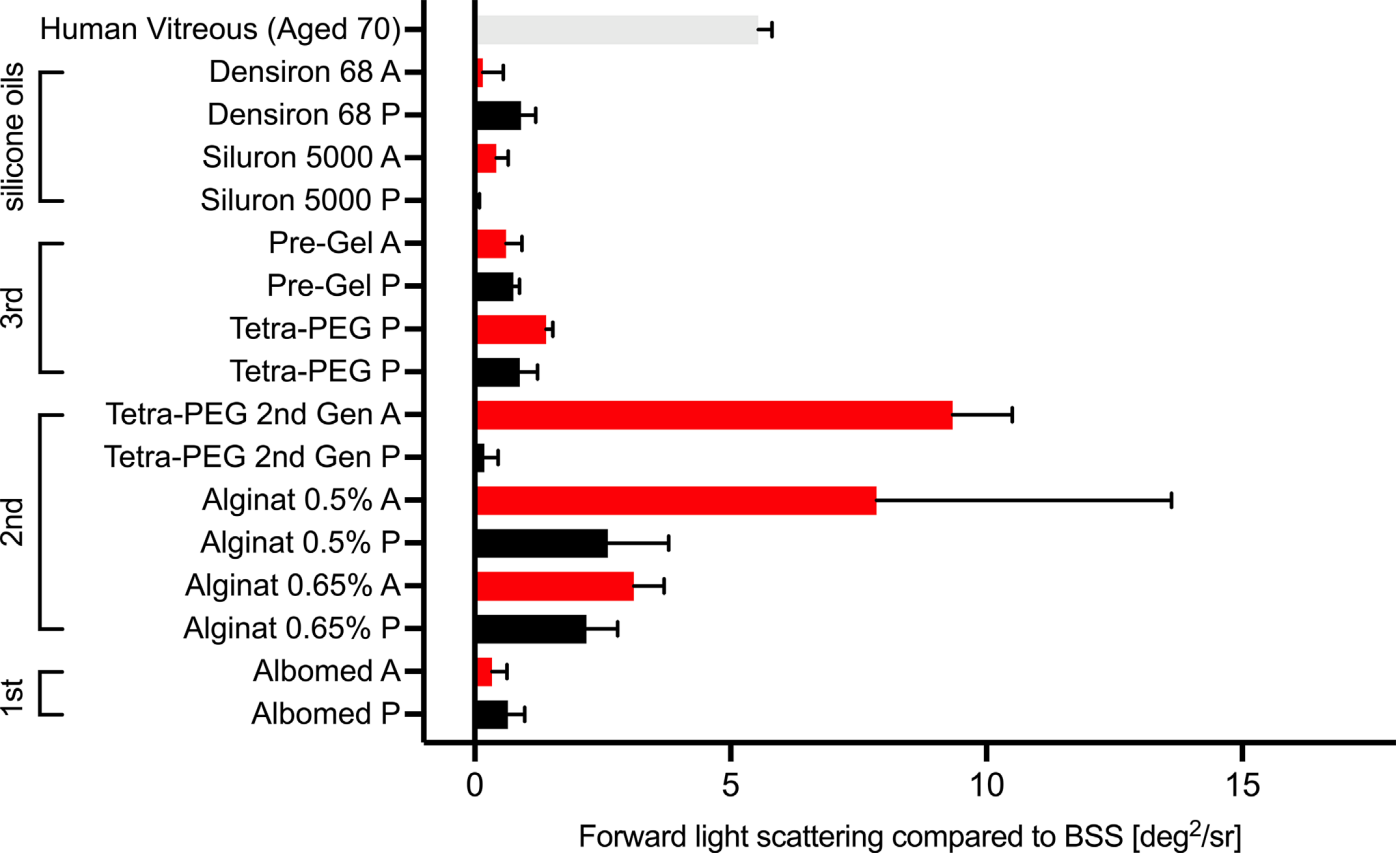
Forward light scattering before and after aging. Two tested second-generation vitreous body replacement hydrogels showed an increase in straylight. Mean ± SEM (N = 3).

Figure 4 depicts the forward light scattering of all vitreous body replacement strategies prior and after aging compared to balanced salt solution. No significant increase in straylight was seen for any of the hydrogels. To simplify comparison between the impact of aging on forward light scattering, second-generation hydrogels were tested after gelation in the testing cuvette. However, when applied clinically, the gel structure gets fragmented because of the injection process through a cannula majorly increasing straylight as previously shown.^26^

#### Viscoelastic Properties

**Figure 5.**
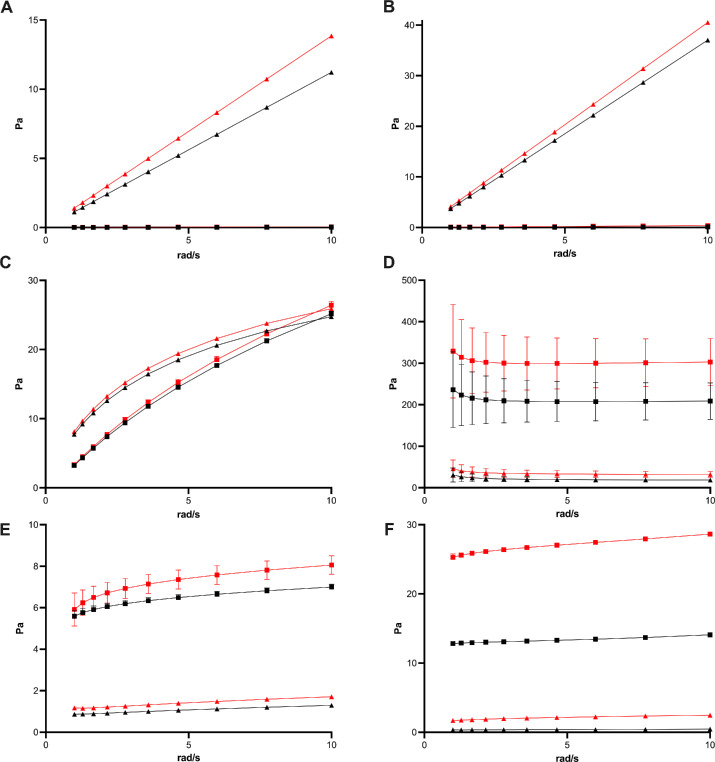
Viscoelastic properties of Densiron 68 (**A**), Siluron 5000 (**B**), first- (**C**: Albomed hyaluronic acid), second- (**D**: alginate 0.5%; **E**: Tetra-PEG), and third- (**F**) generation vitreous body replacement hydrogels before (black) and after (red) aging. Mean and SD of three measurements (SD smaller than symbols, storage modulus [*rectangle*], loss modulus [*triangle*]). A trend toward an increase in the storage modulus for all hydrogels could be explained by the effect of temperature on the hydration of the hydrogels.

Storage and loss modulus before and after aging are depicted in Figure 5. For statistical comparison the loss factor at 6 rad/s was used. No significant difference was apparent for the first-generation vitreous body replacement hydrogel. Although a trend toward a higher storage modulus was seen in all other gels, the loss factor was affected distinctively between gels. The loss factor significantly decreased for the third-generation hydrogel and second-generation hydrogels based on 0.65% alginate, a small but significant increase of the loss factor was apparent for the second-generation Tetra-PEG hydrogel and the 0.5% alginate hydrogel.

## Discussion

In this study, we examined the optical and viscoelastic performance of vitreous body replacement hydrogels after an extended period at 45°C to simulate aging as previously applied to biomaterials in ophthalmology. Additionally, we examined the surgical performance. We can clearly show that most gels that are chemically crosslinked are stable at these temperatures regarding their viscoelastic properties and straylight. Regarding surgical properties, third-generation gels designed to gelate within the vitreous cavity outperformed second-generation hydrogels and currently used silicone oils. Surprisingly, all hydrogels showed a very limited removal speed using the currently clinically used setup.

### Injection Force

The first parameter we evaluated to assess the surgical performance of the hydrogels was the ease of injection. Previously, injection force measurements were used in ophthalmology especially to evaluate the performance of injector systems for intraocular lenses.^43^ Injection force is one of the greatest downsides of high-viscosity silicone oils, one of the currently used standards in clinical practice. The force needed to inject the oils cannot be generated with hand, therefore requiring a pressured setup that allows the injection with a pressure of up to 6 bar. In this study, we evaluated the pressure generated manually by a surgeon. Great differences between the vitreous body replacement hydrogels were apparent. third-generation hydrogels are still in sol-state at injection and undergo sol-gel-transition in situ; thus the needed strength to inject the hydrogels is very low and controllable. In comparison, both the second-generation alginate and Tetra-PEG hydrogels tested in this study were very hard to inject manually. This lowers surgical control and is prone to cause complications during surgery.

### Removal Speed

The option to remove the hydrogels from the vitreous cavity is crucial for their implementation in clinical practice. Many complications can lead to the necessity of hydrogel-removal. To our knowledge, no previous study has ever evaluated the removal of these hydrogels with manual tools typically used in vitreoretinal surgery. The tested third-generation hydrogel showed very similar removal speeds as viscoelastic substances already used during anterior segment surgery. When compared to the aspiration of high-viscosity silicone oil through a specifically designed polyimide-cannula^5^, the third-generation hydrogel can be removed at a faster pace than the oil. Interestingly, the removal is significantly slower than removal of the porcine vitreous body. This is even more pronounced in second-generation vitreous body replacement hydrogels. Vitreous cutters are designed to allow peripheral shaving of the vitreous body close to the retina. Therefore, the cutters are around 20 mm in length. Because hydrogels are viscous, the Hagen-Poiseuille-equation predicts low flow because of the greater length of the pipe and the viscosity of the hydrogels. Regarding the vitreous body, the gel state is achieved by meticulously spaced collagen fibers.^44^^–^^46^ Most likely, this state is disrupted during the process of vitreous removal liquifying the vitreous body and therefore increasing the removal speed. In addition, the removal of the hydrogels is further complicated as no phase segregation from BSS or aqueous humor would occur as it is the case for the currently used lipophilic long term endotamponades, such as the above tested silicone oils. This further complicates the removal process and makes incomplete removal very likely. Better visualization of the hydrogels in the vitreous cavity is therefore crucial. It is unknown whether current techniques used to visualize the vitreous body, such as the injection of triamcinolone or specific vitreous dyes, can be successfully applied for vitreous body replacement hydrogels. Regarding the vitreous body, the gel state is achieved by meticulously spaced collagen fibers.^44^^–^^46^ Most likely, this state is disrupted during the process of vitreous removal liquifying the vitreous body and therefore increasing the removal speed. Although theoretically reducing the amount of cross-linking or polymer concentration in the hydrogels could ease the removal of the substance, all the presented hydrogels already have an ultra-low polymer content. Thus further lowering the polymer content may lead to insufficient gelation and negative effects on the needed viscoelastic properties.

### Straylight

Straylight or forward light scattering represents an option to assess the optical properties of an ophthalmological implant projected onto the retina. This methodology has especially been applied to assess the optical quality of hydrophilic and hydrophobic intraocular lenses^33^^,^^34^^,^^39^ and just recently by our group, to assess the optical quality of vitreous body replacement hydrogels. Prior to this study, it was unknown if extended periods of higher temperatures can cause the hydrogels to opacify, increasing forward light scattering. In this study, we detected very low straylight before aging for all hydrogels. After aging, most gels still did not induce greater amounts of straylight, however the second-generation vitreous body replacement hydrogels based on 0.5% alginate and Tetra-PEG showed greater straylight than what is to be expected from a human vitreous body from a 70-year-old.^26^ Forward light scattering most likely slightly increased in some of the hydrogels due to dehydration of the gel, but all hydrogels still performed well regarding optical properties, and the detected increases are not considered to be clinically relevant. To allow better comparison, we omitted a clinically relevant problem of second-generation hydrogels that must be mentioned: Gel fragmentation during injection. As previously demonstrated,^26^ gel fragmentation introduces new interfaces at which light can be scattered, majorly increasing straylight (Without and with fragmentation 1 deg^2^/sr vs. 12 deg^2^/sr, respectively).

### Viscoelastic Properties

Apart from the first-generation vitreous body replacement hydrogel, all gels showed a differentiated change in their viscoelastic properties. Most gels’ storage modulus increased during aging indicating greater stiffness. Most gels’ loss factor significantly decreased indicating an increase in elasticity and stiffness. This change can be attributed to possible dehydration during the temperature increase. However, these changes to the gel matrix seem to be permanent, because before viscoelastic measurements were reconducted after the aging protocol, the gels were cooled in the same environment to the measurement temperature of 20 °C. This is in line with data from Leon-Cecilla et al that saw an increase in the mechanical robustness of alginate hydrogels that after mechanical dehydration.^47^ The main effects that occur during the heating process of the hydrogels are most likely dehydration, as well as hydrolytic and oxidative degeneration.^48^ Because the reaction components for both the second-and third-generation Tetra-PEG hydrogels were calculated to allow for a complete conversion, no further cross-linking reactions are expected to occur during the heating process. The cross-linking reaction of the alginate hydrogel is based ionic cross-linking. Therefore dehydration effects may take place during the aging process caused by a loss of calcium through accelerated diffusion. Consequently, a higher temperature more likely leads to less cross-linking of the alginate hydrogel. Applying heat is one way of accelerated aging commonly used for biomaterials. Especially in the context of the intraocular use where a high shear stress caused by saccadic eye movements is applied, another methodology to induce aging would be shear stress and angular acceleration, which should be applied in further studies.

However, even after aging, the presented viscoelastic properties still resemble the viscoelastic properties of the vitreous body. This stands in great contrast to the viscoelastic properties of the oils (Figs. 5A, 5B), which are currently clinically used.

### Limitations

This study shows minor limitations. The applied aging protocol is tested on other ophthalmic implants, more specifically intraocular lenses based on acrylic polymers. Research validating this procedure for vitreous replacements has yet to be conducted and also other protocols should be applied in the future. However, this study is a first starting point in assessing vitreous body replacement hydrogels systematically in laboratory testing. Additionally, surgical testing was performed in vitro. Further studies should investigate injection and removal speed in in vivo experiments.

## Conclusions

In conclusion, this study demonstrates that no clinically relevant change of the viscoelastic and optical properties of vitreous body replacement hydrogels occurs when stress-tested under an accelerated aging protocol previously applied for ophthalmological implants. Additionally, this study demonstrates surgical weaknesses of the hydrogels that need to be addressed before widespread clinical use, namely a low removal speed. Pre-linked hydrogels, so called *second-generation vitreous body replacement strategies*, showed inferior performance regarding surgical properties.
